# Feeding kinematics of a surgeonfish reveal novel functions and relationships to reef substrata

**DOI:** 10.1038/s42003-023-05696-z

**Published:** 2024-01-03

**Authors:** Michalis Mihalitsis, Peter C. Wainwright

**Affiliations:** Department of Evolution and Ecology, University of California, Davis, CA 95616 USA

**Keywords:** Biomechanics, Behavioural ecology, Evolutionary ecology, Animal behaviour, Ichthyology

## Abstract

Biting to obtain attached benthic prey characterizes a large number of fish species on coral reefs, and is a feeding mode that contributes to important ecosystem functions. We use high-speed video to reveal the mechanisms used by a surgeonfish, *Acanthurus leucosternon*, to detach algae. After gripping algae in its jaws, the species pulls it by ventrally rotating both the head and the closed jaws, in a novel use of the intra-mandibular joint. These motions remain in the plane of the fish, reducing the use of a lateral head flick to detach the algae. The novel ability to bite and pull algae off the substrate without bending the body laterally minimizes exposure to high water flows, and may be an adaptation to feeding in challenging reef habitats such as the crest and flat. Therefore, our results could potentially represent a key milestone in the evolutionary history of coral reef trophodynamics.

## Abstract (web summary)

High-speed video of feeding in a surgeonfish reveals novel functions, and suggests an adaptation to feeding in high flow environments, as well as how fish interact with the substrate

## Introduction

The long history of research on the functional morphology of feeding in fishes has emphasized the importance of suction during prey capture^1–3^. High-performance suction feeding has been linked to the explosive success of ray-finned fishes, the most diverse lineage of aquatic vertebrates^3–5^. In spite of the ubiquity of suction feeding among fishes, the use of direct biting to remove prey attached to a substrate has emerged as another key, albeit less studied, feeding mechanism that is particularly important on coral reefs where approximately 40% of species feed by biting attached prey^6–8^. Fishes have interacted with benthic substrata for millions of years (potentially up to 230 Ma), resulting in the co-evolution of fishes and reefs^9^. If we are to understand the evolution of coral reef ecosystems, the most biodiverse systems on Earth today, we must also understand the mechanisms and behaviours that fish use to remove these benthic resources.

Among the most ecologically important reef biters are herbivores, being capable of profoundly influencing benthic communities by removing competitively superior algae^10^. Indeed, herbivores are directly linked to the resilience of reef systems under threats due to climate change^11^. While the ecological dynamics of herbivory have been a focus for decades^12–15^, the functional morphology of feeding on algae has only been sparsely studied. Surgeonfishes are some of the most abundant coral reef herbivores^16,17^, and are known to have a strong relationship with algal turfs^18^, the most abundant benthic resource in coral reef environments^19^. Despite their crucial ecological role in these ecosystems, little is known about how these fishes feed, specifically, how they are able to detach algae from these environments. Perevolotsky, et al. ^20^ recently showed a mechanism through which two surgeonfish species from the genus *Zebrasoma* use their entire body and fins to detach prey from benthic substrates through the use of a lateral head-flick. It is, however, still unclear how widespread this behaviour is among surgeonfishes and whether other species use other mechanisms when feeding on algae.

The kinematics and anatomy of biting have previously been investigated in other surgeonfish species^20–22^ as well as other fish lineages^23–26^. Interestingly, a primary finding of these studies has been the independent evolution of an intramandibular joint (IMJ), which refers to a secondary joint in the lower jaw between the dentary and articular bones. While the IMJ is a functional innovation associated with the feeding mode of biting^27^, the function attributed to the IMJ is inconsistent. Thus far, its function has been described as providing enhanced gape expansion^21,24,27^, increased force production onto attached prey^24^, enhanced ability to close the jaws when they are extended^28^, and allowing the modulation of tooth orientation^23^. Overall, the IMJ appears to be multifunctional, promoting multiple possible advantages during feeding on attached prey.

In this study we investigate algae cropping in the surgeonfish, *Acanthurus leucosternon*, using high-speed filming and anatomical analyses to determine the role of specific anatomical components during feeding (jaws, neurocranium, pectoral girdle, pectoral fin, pelvis, and posterior end of body). In so doing, we identify novel behaviours and mechanisms, including an IMJ with a previously undescribed function. Furthermore, we show the co-option and novel use of movements traditionally used for suction feeding in teleosts, to have an altered function during biting in *A. leucosternon*.

## Results

**Fig. 1:**
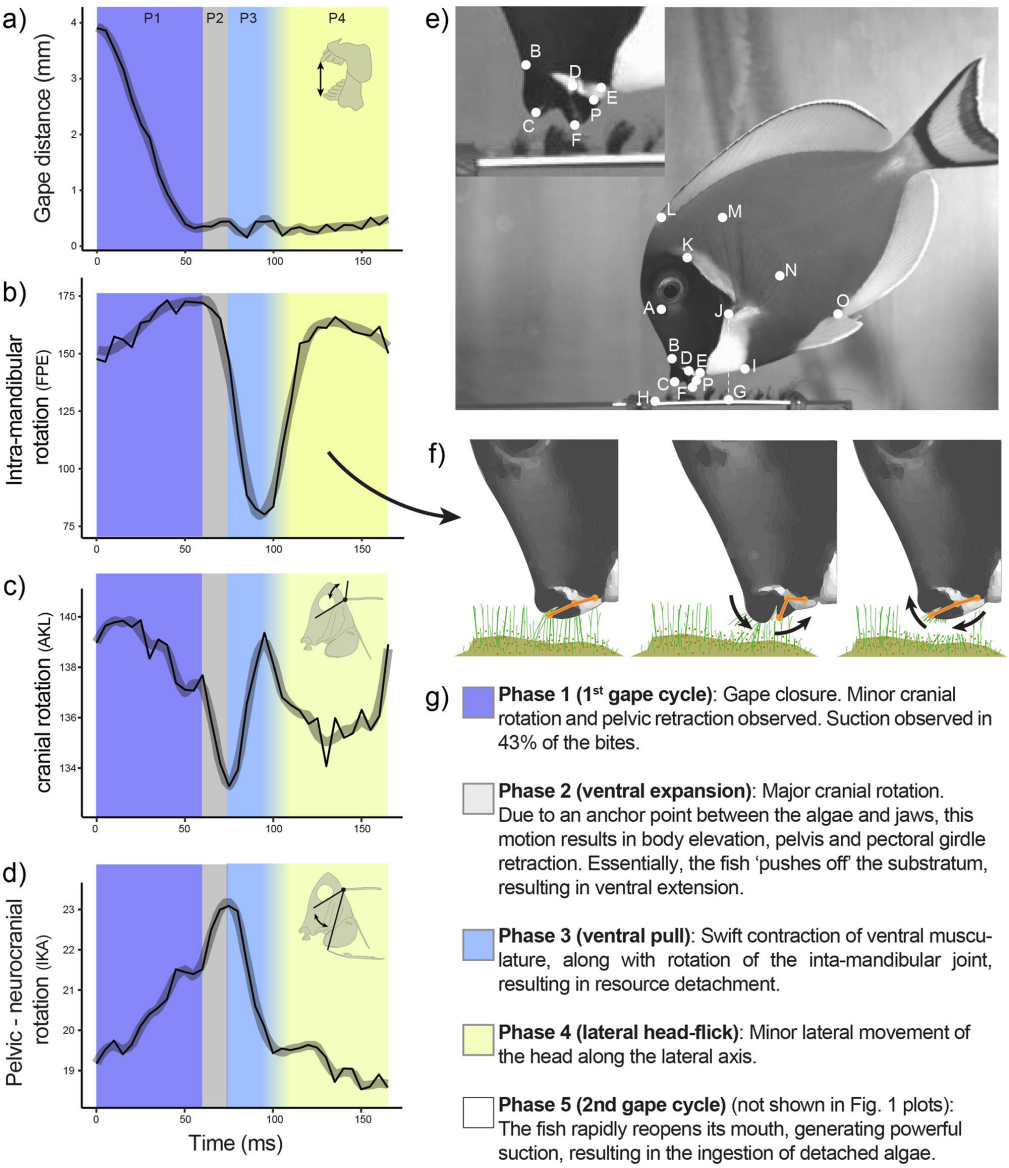
Kinematic profile of the surgeonfish, *Acanthurus leucosternon*, grazing on benthic algae (see also Supplementary Movies 1, 2). **a** Gape distance over time, (**b**) Jaw rotation through the intra-mandibular joint over time (angle between landmarks FPE). **c** cranial rotation over time (angle between landmarks AKL), (**d**) pelvic-neurocranium rotation over time (angle between landmarks IKA), (**e**) photo of an *A. leucosternon* with all the landmarks analysed throughout the study, (**f**) visual representation of how the intra-mandibular joint is used to detach filamentous algae, (**g**) descriptions of the 5 phases taking place throughout a bite.

We found that during feeding on algae, fish did not contact the underlying substrate with their teeth, but directly bit the algae. Each bite consisted of an initial jaw opening and closing around the filamentous algae (first gape cycle), often accompanied by a distinct suction event that drew the algae into the mouth as the jaws closed around it. Once the jaws were closed around the algae the fish pulled it ventrally, through a swift ventral rotation of the closed jaws. As described in detail below, this ventral rotation involved both ventral cranial rotation as well as ventral rotation of the closed jaws on the anterior end of the neurocranium (Fig. 1, see also Supplementary Movie 1). This previously undescribed dorsal-ventral movement of the closed jaws is made possible by flexion of an intramandibular joint (IMJ) between the articular and dentary bones, movement at the quadrate-articular joint and the upper jaw sliding a short distance ventrally or dorsally on its articulation with the anterior process of the neurocranium (Fig. 1, see also Supplemental Movie 2). As the neurocranium was flexed ventrally, both the lower and upper jaws rotated ventrally so that the jaw tips moved in the same direction as the neurocranium, while remaining closed and holding on to the algae. This ventral pull was followed by a subtle lateral head flick as the fish pulled away from the substrate, leading to a second gape opening and closing cycle, during which the detached algae held between the teeth was ingested through a second suction event. The duration with which the mouth opened for the 1st suction event (120 ms ± 10.7 standard error) of the bite was found to be significantly different, and 7.6 times higher than the duration in the 2nd suction event (15.7 ms ± S.E.) (Supplementary Fig. 2, GLS, *t* = −9.7, *p* < 0.001). The full bite sequence had a duration of less than half a second (416.8 ms ± 17.4 S.E.). Based on the key motions described above, we divided the bite sequence into five phases: (1) gape closure, (2) ventral expansion, (3) ventral pull, (4) lateral head-flick, and (5) second gape cycle.

**Fig. 2:**
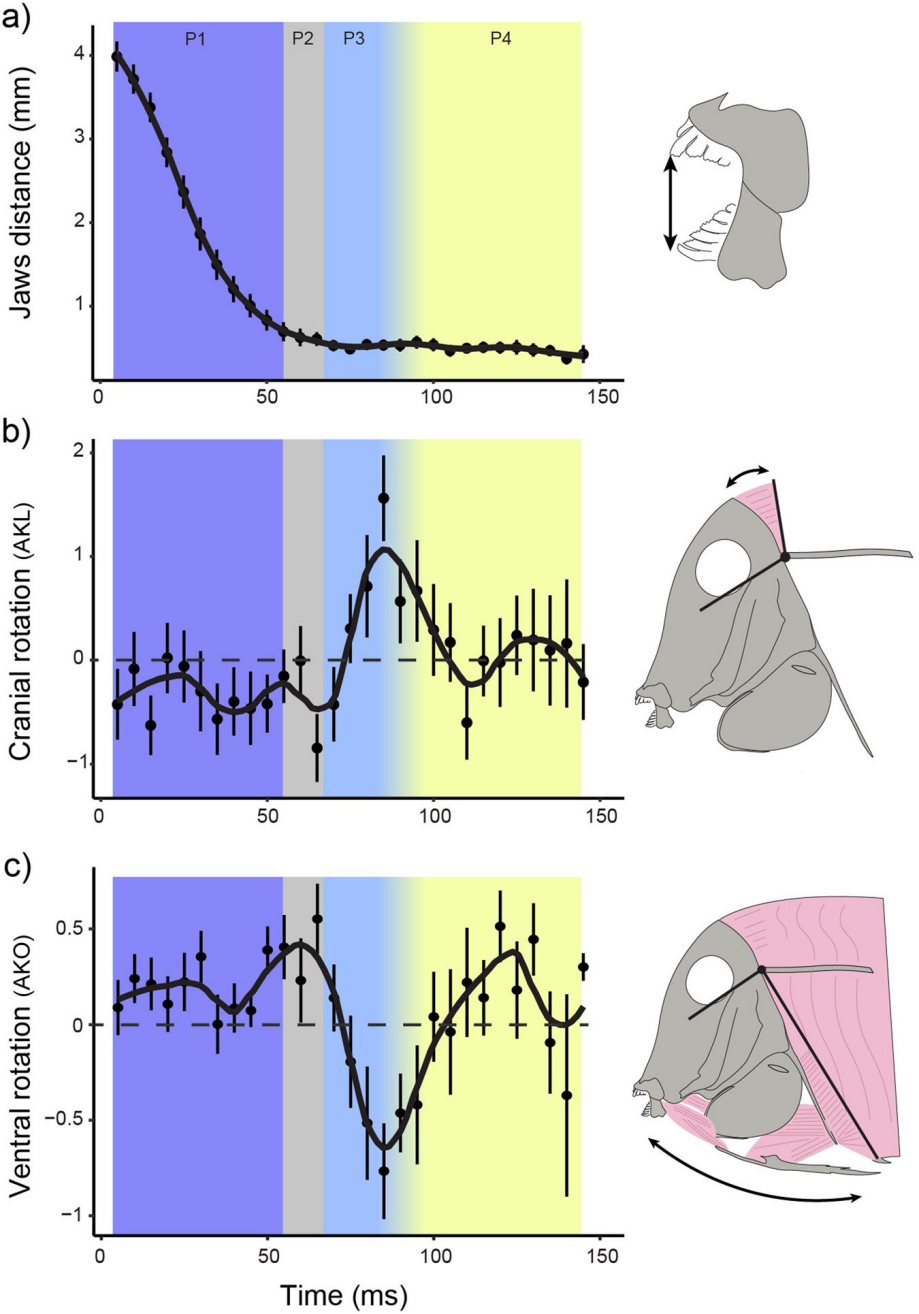
Mean kinematic profiles of key functional components for all bites (*n* = 23). **a** shows the distance between the tips of the upper and lower jaws (mm). Plots **b**, **c** show the mean amount of angular change that takes place between timesteps (angle_t_- angle_t -1_). **b** shows the amount of change in cranial rotation that occurs, (**c**) shows the amount of change in ventral rotation that occurs in each timestep. Point ranges represent the mean and standard error for all bites, at a given timestep.

**Fig. 3:**
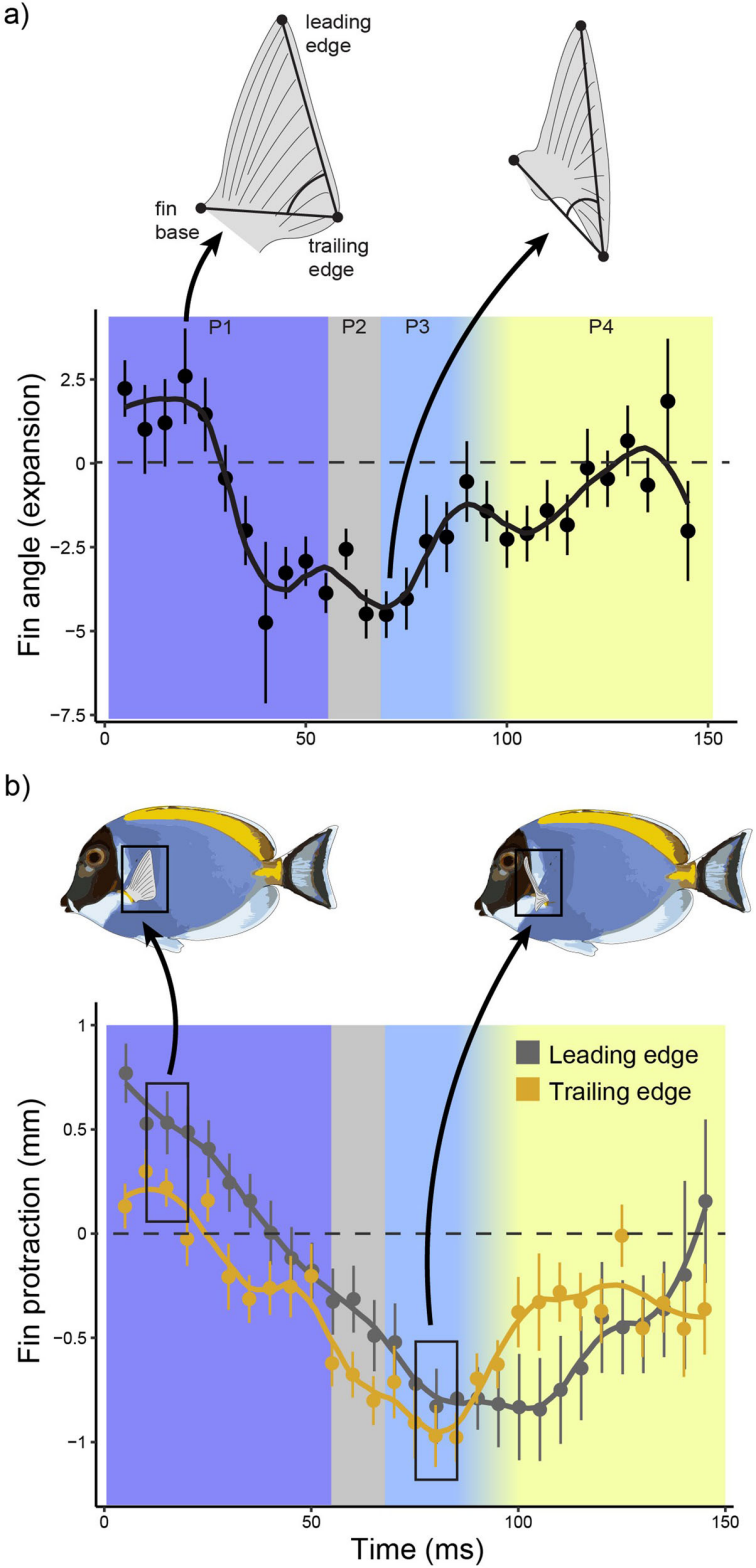
Pectoral fin kinematics of *Acanthurus leucosternon* during feeding (see also Supplementary Movie 1) (*n* = 23). **a** Angle between the leading edge, trailing edge, and base of the pectoral fin. Throughout the bite duration, the fish spreads its pectoral fin, resulting in an angle decrease, and (**b**) pushes the fin anteriorly (protraction). Point ranges represent the mean and standard error for all bites, at a given timestep. The *x*-axis shows time (ms), whereas the *y*-axis shows the amount of change relative to the previous timesteps. Values above 0 represent an increase in fin angle (**a**) or fin protraction (**b**), whereas values below 0 represent a decrease in fin angle (**a**) or fin protraction (**b**).

Phase 1 (gape closure) refers to the jaw closure as the fish descends on the benthos (Figs. 1a,  2a). During jaw closure, cranial elevation (relative to the fish body) (angle AKL in Fig. 1e) of 5.3 degrees (±0.6 S.E.) was observed, along with 1.8 degrees (±0.2 S.E.) of pelvic rotation (angle AKI in Fig. 1e) (Fig. 1, Fig. 2). During phase 1, the pectoral fins start to rotate from a posterior-dorsal posture to an anterior-ventral position (Fig. 3a). For 48% of the bites analysed, suction was observed as the jaws closed on the algae (more on this below).

**Fig. 4:**
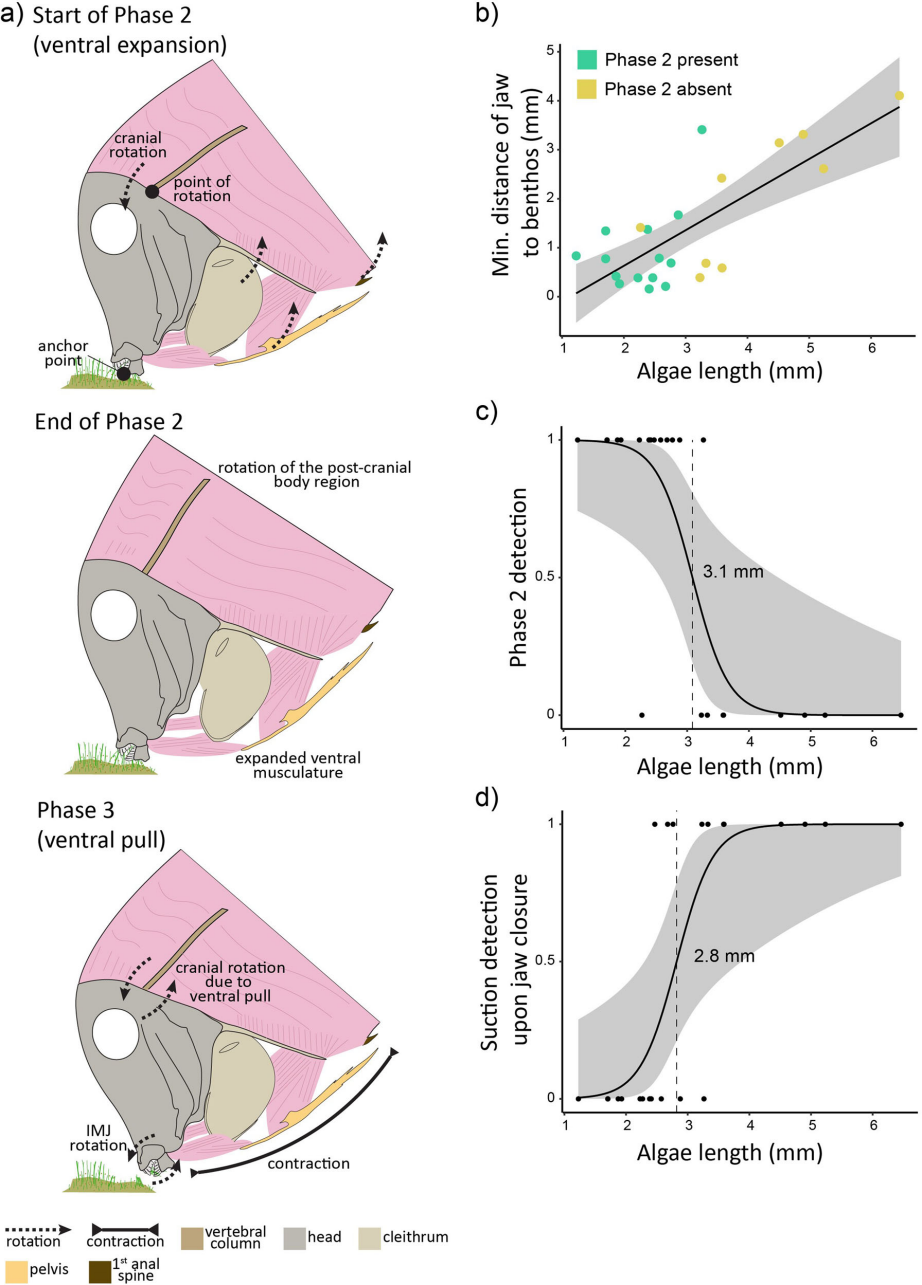
The relationship between algal length and bite kinematics (*n* = 23). (**a**) shows a visual representation of Phase2 and Phase 3, along with the ventral musculature and bone elements (see also Supplemental Movie 1). (**b**) shows the minimum distance the lower jaw reached during the bite relative to the benthos (mm). (**c**) shows the odds of detecting the Phase 2 behaviour (i.e., fish pushing away from algae using the benthos). A value of 1 represents the presence of Phase 2 in the bite, whereas a value of 0 represents the absence of it. The odds at which this relationship shifts from zero to one (i.e., odds = 0.5) is at an algal length of 3.1 mm. (**d**) shows the odds of detecting suction during jaw closure, relative to algal length. A value of 1 represents the presence of suction in the bite, whereas a value of 0 represents the absence of it. The odds at which this relationship shifts from zero to one (i.e., odds = 0.5) is at an algal length of 2.8 mm.

Phase 2 (ventral expansion) starts when the fish has closed its jaws around the algae, and ends with the initiation of a ventral pull (Fig. 1). During phase 2, which lasted about 19.6 ms (±1.7 S.E.), cranial elevation of 4.2 degrees (±0.5 S.E.) was observed, along with 1.8 degrees (±0.3 S.E.) increase in the angle between the neurocranium and the pectoral girdle of the fish (angle AKJ in Figs. 1e), and 1.8 degrees (±0.2 S.E.) increase in the angle between the neurocranium and the pelvis of the fish (Figs. 1c, d, 2c). During this phase, the neurocranium remained in a relatively fixed position with respect to the substrate, being stabilized by attachment to the benthos through the jaws gripping the algae (anchor point), while the body, the pectoral girdle, and the pelvis, rotate dorsally about the craniovertebral joint through cranial rotation (Figs. 2, 4a). During this phase the pectoral fins continued to rotate towards an anterior-ventral position. The trailing edge of the pectoral fin began to protract (Fig. 3b). This phase was observed in 61% of the bites analysed. For the remaining bites, phase 1 was directly followed by phase 3.

**Fig. 5:**
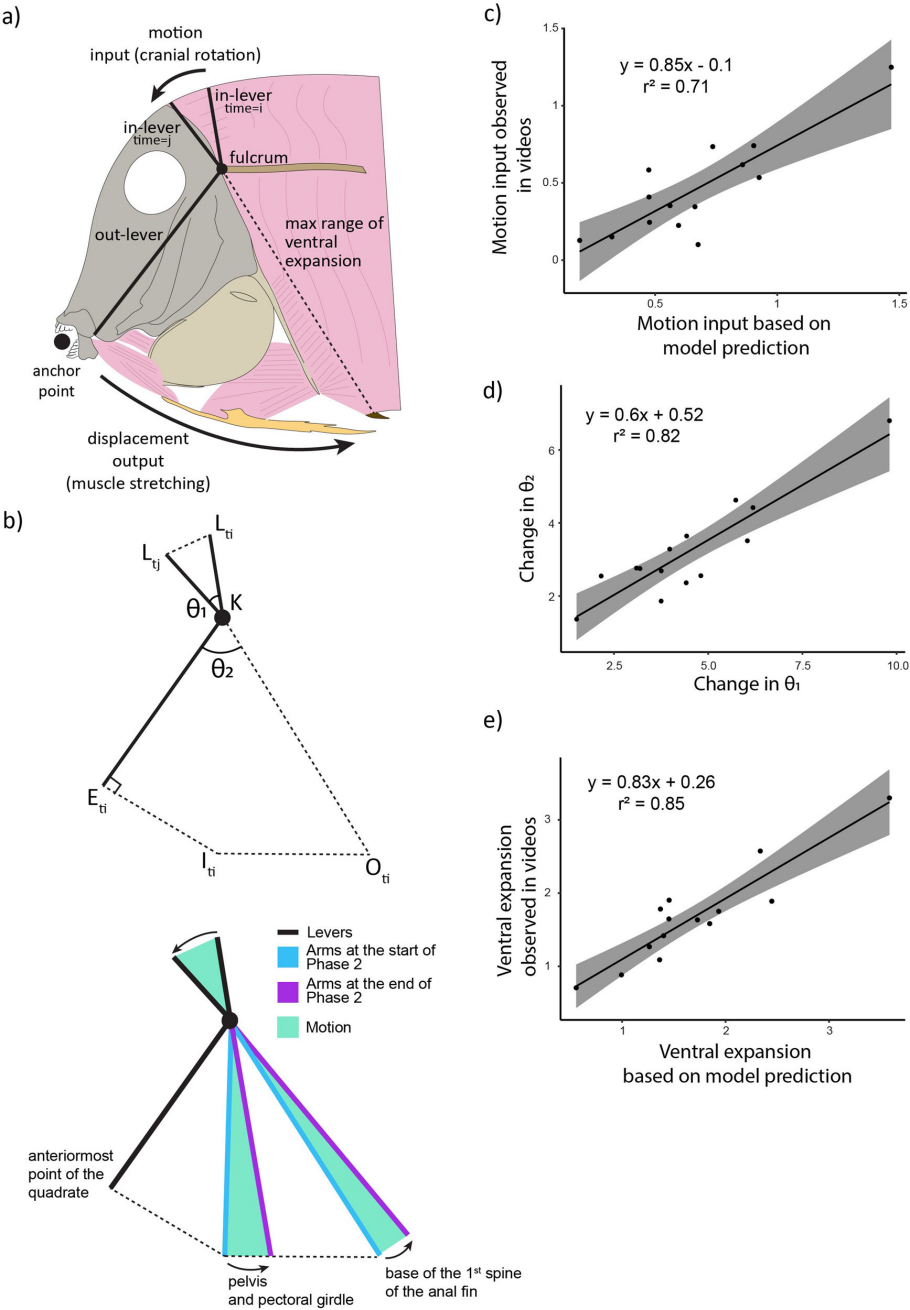
Biomechanical interpretation predicting the functional feeding traits increasing the performance of the ventral pull (Phase 2) of *Acanthurus leucosternon* (*n* = 14). **a** Illustration of *A. leucosternon* osteology and myology relating to the proposed system, (**b**) proposed biomechanical interpretation with cranial rotation acting as a motion input, and the ventral side of the fish acting as a motion output system. Letters represent the corresponding landmarks, whereas θ represents the angles between the motion input and output, and *t*_i_ and *t*_j_ represent timesteps. **c** Motion input based on the proposed model against the motion input observed in the landmarked videos (distance covered by L throughout Phase 2). **d** Amount of change in θ_1_ observed in videos, against the motion output (θ_2_) observed in videos. **e** Ventral expansion (sum of distances EI & IO) predicted by the model, against the ventral expansion observed in videos.

Based on analysis of videos and anatomical observations, we propose a biomechanical interpretation that links cranial rotation to the ventral expansion seen in Phase 2 (Fig. 5). Here, ‘cranial rotation’, occurring while the head is stabilized by attachment to the benthos by holding onto the algae with the teeth, results in dorsal rotation of the posterior body regions and, hence, ventral expansion, during which ventral muscles are stretched. The skull of *A. leucosternon* shows less kinesis than many other teleosts so dorsal flexion of the body on the neurocranium is mostly transmitted directly through the skull to ventral components. We found that this model accurately predicted the output motions using input cranial rotation measured from the videos (GLM; *t* = 5.5; *p* < 0.001, Fig. 5c) (angle AKL). The angle change from cranial rotation (θ_1_), was significantly correlated with the sum of the angle outputs (θ_2_ + θ_3_) (angles IKE and OKI) based on our videos (GLM; *t* = 7.35; *p* < 0.001, Fig. 5d). Based on these results, we then measured the levers KE, KI, and KO on each of the specimens from our videos, and based on trigonometric relationships calculated the change in distance predicted between EI and IO from the motion outputs. These changes in distance predicted from our model were strongly correlated with the changes in distance observed in our videos for both EI (GLM; *t* = 11.3, *p* < 0.001; Fig. 5e), as well as OI (GLM; *t* = 5.7, *p* < 0.001; Fig. 5f). Overall, we found high agreement between the predicted (based on model) and observed (based on videos) sources of motion.

Phase 3 (ventral pull) is initiated through swift ventral contractions between the neurocranium and the pelvis (presumably through contraction of the protractor hyoideus and sternohyoideus) (Fig. 1c, d, Supplementary Fig. 3a), between the neurocranium and the pectoral girdle (Supplemental Fig. 3b), and between the pectoral girdle and the rest of the body (probably through hypaxial musculature attached to the postcleithrum and other ventral regions of the pectoral girdle). During this ventral cranial rotation both the upper and lower jaws remain closed and simultaneously rotate ventrally (relative to the neurocranium), a motion that involves mid-mandible flexion at the intramandibular joint between the dentary and articular, so that the anteriormost part of the jaws move to a position a few millimetres away from their starting position (Fig. 1b, f). Flexion at the IMJ was considerable, reaching up to 93 degrees of rotation (Fig. 1b). This combined action of ventral rotation of the neurocranium and ventral rotation of the jaws was used to detach the algae, and is followed by Phase 4, where the ventral pull changes more to a subtle, laterally oriented head flick.

During phase 5, a second gape cycle occurred. Jaw opening was significantly faster than the first jaw opening, lasting on average 15.7 ms (±0.7 S.E.), thus making it on average 7.6 times faster than the first jaw opening. We found that this second jaw opening always was accompanied by a burst of suction, whereupon algae was moved rapidly from the jaws towards the buccal cavity of the fish.

Both the propensity of fishes to exhibit suction during the initial bite, and the use of Phase 2 before pulling on the algae, depended on the length of the algae they fed on (Fig. 4b–d). The probability of observing suction during the initiation of the bite rose about 0.5 at an algal length of 2.8 mm (Fig. 4d; GLM, *z* = 2.3, *p* < 0.05). The probability of a distinct Phase 2 rose to 0.5 at an algal length of 3.1 mm (Fig. 4c; GLM; *z* = −2.249, *p* < 0.05). Shorter algal lengths were associated with the fish extending its jaws closer to the substratum during the bite (GLM, *t* = 5.471, *p* < 0.001; Fig. 4b).

## Discussion

Our study provides a detailed analysis of the feeding functional morphology and kinematics of the surgeonfish *Acanthurus leucosternon*. We identify a novel set of behaviours and mechanisms used when this species feeds on benthic algae. In all feeding sequences, *A. leucosternon* made use of its intra-mandibular joint functions during biting. Flexion of the intra-mandibular joint allowed the fish to ventrally rotate the closed upper and lower jaws, pulling on the algae in its grip. Ventral rotation of the closed jaws was accompanied by ventral rotation of the head and the combination of these two movements was used to break the algae free from the substrate. There are two important points to be made about the kinematic profile of *Acanthurus leucosternon*. First, this is the first report in a benthic feeding reef fish of the use of either ventral head rotation or ventral jaw rotation to pull attached prey away from their holdfast. Second, as we discuss below, the fact that these motions, ventral head rotation and ventral jaw rotation, stay in the major plane of the fish’s body may have major implications for the ability of this species to feed in high flow habitats on the reef.

### Novel function of an intra-mandibular joint, used in ventral pulling

The diversity of functions previously ascribed to the IMJ in different benthic feeding fishes is striking. While it is noteworthy that the addition of a joint within the mandible can provide numerous advantages to benthic feeding fishes, it raises the question of why different lineages seem to emphasize different functions of the IMJ. A key to this variation may be that benthic resources display a large diversity of structural integrity, consistency, hardness, and other properties, while the specific mechanisms that fish use to dislodge benthic prey also vary. For example epilithic filamentous algae is removed through cropping in many surgeonfishes^29–31^, whereas endolithic algae is obtained through scraping by some parrotfish^32–34^. Even within a single algal plant, different fish species may feed on different parts (stalks vs. leaves)^12^, presumably encountering differences in the structural integrity of the different plant tissues. There is considerable diversity in the mechanics needed to remove algae, and if we consider other benthic resources on reefs (e.g., corals, mucous, detritus, sponges) it is even easier to see that the mechanical challenges are diverse. IMJs have evolved at least eight times in reef fishes and although the specific function of the novelty varies, it is always associated with benthic feeding^27,35^. It therefore appears that one of the benefits of the IMJ is its versatility. We add to the list of IMJ functions, demonstrating a novel function of an IMJ, used to allow ventral rotation of the closed jaws while detaching epilithic filamentous algae. These observations suggest that future kinematic studies of benthic feeding should carefully consider the nature of the feeding treatment used in their study. Treatments such as gel blocks or agar plates may not produce the same kinematic patterns as natural foods, potentially obscuring the realized function of the anatomy and behaviour of the organisms studied.

While an IMJ has also been reported in other surgeonfishes^21,27^ it is not yet known how widespread this trait is among acanthurids or how it functions in those species. It is noteworthy that for other surgeonfishes for which benthic feeding kinematics have been reported, some use distinct lateral head flicks to dislodge algae^20^. Flexion in the IMJ operates synergistically with ventral head rotation to detach algae in *A. leucosternon*. These motions likely reduce reliance on the lateral head flick, which was typically quite subtle, in contrast to the sharp movements used by species within *Zebrasoma*^20^. This surprising diversity in the few acanthurid species that have been studied raises the possibility that other kinematic and functional morphological novelties may be found among the many benthic-feeding species in this family. We note that the ventral pulling behaviour in *A. leucosternon* is cryptic and not readily observed without the benefit of high-speed video. There is therefore an urgent need to study surgeonfishes more broadly to understand the diversity of their feeding mechanisms and behaviour as this may provide insights into the considerable ecological diversity found in the family.

### Linking cranial rotation to ventral expansion. A new way of interacting with the benthos?

Epaxial muscles in teleosts typically function during feeding to elevate the neurocranium during suction strikes, providing a key source of power for rapid buccal expansion^36,37^. In our study, cranial elevation was not observed during the bouts where gentle suction was observed as the species approached the algae. Nevertheless, cranial elevation is of central importance to the feeding mechanism of this species. In an interesting departure from the widespread use of cranial rotation in suction feeding, we found that it was used as a preparatory motion to stretch ventral structures, as well as being employed as one of two key movements to pull algae away from the substrate. During the initial stretching movement, the neurocranium was stabilized by the jaws gripping the algae, so that flexion of the cranio-vertebral joint resulted in the post-cranial body of the fish rotating dorsally, thus stretching the ventral regions of the fish (Figs. 2b, c,  4a). Based on the ventral anatomy of the fish, this motion appears to stretch the ventral muscles, including the protractor hyoideus and sternohyoideus between the dentary, hyoid bar and pectoral girdle; hypaxialis muscle between the pectoral girdle and posterior body regions. Stretching potentially acts as a preparatory movement prior to tearing the algae from the substratum. It may aid the fish in the subsequent pulling motion by separating ventral elements so that muscles are able to operate on more of their full range of motion (however, the potential presence of an elastic recoil effect would need to be tested). We therefore highlight this example of a widespread mechanism that plays a central role in suction feeding by other teleosts, being co-opted in this surgeonfish for a fundamentally different feeding function. One key to this novelty is the stabilization of the neurocranium as the fish bites the algae attached to the benthos, so that cranial rotation results in dorsal rotation of the postcranial body region, as opposed to cranial elevation. In other words, the fish uses the algae and substrate to modify the function of cranial rotation.

When modelling this motion, we describe cranial rotation as an input motion, resulting in the expansion of ventral musculature (Fig. 5). In this system, the length of the out-levers that extend from the rotation point of the neurocranium determine the amount of motion at the tips of these structures. For a given amount of cranial rotation, the long out-lever results in considerable ventral motion of the jaw joint (Phase 2), leading to greater displacement of the jaws (Phase 3). As these out-levers are generally a function of the dorsal-ventral depth of the head and body, the more deep-bodied a fish is, the more motion is achieved through this mechanism. Coral reef fishes extracting resources from the substratum (i.e., biters) contain some of the most iconic coral reef fishes (e.g., Acanthuridae, Siganidae, Chaetodontidae, Pomacanthidae). These lineages are characterized by deeper body shapes compared to other groups^6^. While having a deep body has previously been linked to high manoeuvrability^38,39^, and anti-predatory mechanisms^40–42^, our observations suggest there may also be a consequence for benthic feeding performance. This appears to be an additional way in which benthic biters can take advantage of their unusual body shape.

Pectoral fin movements are known to be an integral component of benthic biting kinematics in fishes^20,26^. Our observations are similar to previous studies, where we found that pectoral fin protraction likely functions to aid in the detachment of prey or as stabilizers that help the fish remain in position during the ventral pull. The mechanism through which this may occur, is by the fish counteracting the ventral movement of the neurocranium and rest of the body. In other words, the spreading and protraction of the pectoral fins (movement in the opposite direction of the ventral pull), may aid the fish in holding its position and thus take multiple bites per feeding bout. Essentially, our results show that the pectoral fin anatomy may not only be linked to swimming, but feeding abilities as well, by generating thrust in the opposite direction of the ventral contraction (Phase 2), presumably increasing the forces applied onto the algae, providing stability, or a combination of both.

### Links to coral reefs: ecological and evolutionary implications

Shallow coral reef habitats are often characterized by high, ever-changing current speeds and strong wave action^43^. These high-energy habitats are the feeding zone of a restricted group of species that are able to access these areas by virtue of their swimming performance^44–46^. Feeding benthically in habitats with extreme flows may present particular challenges to an algae-grazing fish that relies on lateral head flicks to dislodge algae. Multiple studies have found for areas with strong currents, fishes will turn to face oncoming currents (i.e., rheotaxis)^47–49^. Furthermore, experimental studies have shown that under stronger currents, fishes decrease the number of strikes with a lateral component, attributing this to being able to remain stable in the water column, as well as reducing energy expenditure^47,49,50^. Surgeonfishes, and other biters which employ a sideways head-flick when detaching algae may expose their bodies to high destabilizing forces in the presence of onrushing currents. Use of the IMJ and ventral body contractions to pull algae ventrally when detaching them, allows the key feeding motions to stay in the same plane as the body of the fish, avoiding the lateral head motions that would increase the surface of the fish exposed to an onrushing flow of water. By minimizing the need for lateral head flicks, the IMJ and ventral contractions may represent an adaptation to algae cropping in high flow habitats. In other words, while previous work on IMJs has focused on the trophic diversity they promote (‘how’ and ‘what’ they feed on), we suggest that IMJs may also diversify the locations the fish may feed at (‘where’ they feed).

The reef crest and flat have been shown to have the highest rates of herbivory on coral reefs^16,51^. The average turf length on reef crests is about 3 mm, with turf lengths in other zones being significantly greater^52^. Furthermore, turfs on the crest are the most productive, low on sediment, and high on detrital material, thus making them highly desired by grazing reef fishes such as surgeonfishes^53^. Given that *A. leucosternon* only employed Phase 2 when feeding on algae under 3.1 mm, the use of Phase 2 motions would be advantageous to feeding on the reef crest. Indeed, the species is predominantly found feeding in shallow, high-flow habitats on coral reefs, such as the crest and flat^54–56^. In their study of the relationship between algal turfs and reef topography (elevation and surface angle), Tebbett, et al.^57^ found that for the shallowest reef habitats included in their study, the algal turf length was approximately 3 mm. This suggests that the motions quantified throughout Phase 2 may be an adaptation to feeding on the reef crest and other similar habitats.

## Material and methods

### Filming and kinematic descriptions

Individuals were sourced from commercial vendors. A total of five individuals were dissected and quantified with regards to their anatomy. Of those, three individuals (Standard Length: 83.6, 85.5, 77.3 mm) were also recorded feeding, using a high-speed camera (Fastec Imaging, High Spec 2G) at 1000 frames per second (fps). Two strobe lights were placed in front of the filming tanks that were maintained at 22-23^o^C at approximately an angle of 45^o^. Individuals were filmed feeding on filamentous algae to mimic algal turfs as close as possible (see Supplemental Fig. 1). The algae was sewed into custom-made feeding plates with equally distanced and sized holes through which the algae was threaded, thus minimizing for potential differences in the algae volume removed per bite (see Supplemental Fig. 1). For distance calibration, a ruler was placed at the location of the feeding treatment prior to recording feeding sequences. The fish were presented with the feeding treatments and filming was commenced. Only footage where the fish appeared to be in lateral view to the camera were used for subsequent analyses. If throughout the bite sequence the fish altered its orientation to a non-lateral state, landmarking was ceased at that frame. Filming ended when the fish appeared to be satiated, which usually lasted about 15 min per individual for each filming session. Videos were then extracted to the software ImageJ, where sequences were cropped between the frame where initial mouth opening occurred and at the end of the final jaw closure, after the fish pulled away from the substrate following the bite. A total of 23 bites were digitized with landmarks (*n* = 8, 10, and 5 respectively from each individual). All experiments were carried out in accordance to the University of California, Davis Institutional Animal Care and Use Committee, protocol number: 22206.

### Data analysis

Once imported to ImageJ, landmarks were placed in 15 locations on the body of the fish, as well as the benthos, to extract kinematic traits (for an overview of landmark positions, see Fig. 1e) throughout the feeding sequence of the 23 feeding sequences. The length of the algae targeted at each bite taken was also measured. Landmarks were placed every 5 frames (5 ms), resulting in a mean of 1041 (±50.5 S.E.) landmarks placed per feeding sequence (or approximately 24,000 landmarks placed in total). For a detailed description of the landmarks placed in the sequences, see Supplemental Table 1. Landmark coordinates were then imported into the software R^58^ for further analysis. A kinematic profile was generated for each individual bite. This profile tracked rotational changes to a series of angels calculated between three landmarks, which captured key motions occurring during the bites. Movements of individual landmarks through time were found from the following equation:

1 $$d=\sqrt{{({x}_{t}-{x}_{t-1})}^{2}+{({y}_{t}-{y}_{t-1})}^{2}}$$

where ${x}_{t}$ and ${y}_{t}$ represent the *x* and *y* coordinates of a landmark at a given time (*t*). The distance between two separate landmarks was also calculated, to investigate changes in the relationship between different components on the body of the fish through the sequence. This was done using the following equation:

2 $$d=\sqrt{{({x}_{{it}}-{x}_{{jt}})}^{2}+{({y}_{{it}}-{y}_{{jt}})}^{2}}$$

where ${x}_{{it}}$ is the x coordinate of a landmark $i$ at a time $t$. We defined *t* = 0 in all our kinematic profiles as the first frame in which the opened jaws began to close. This was done using the equation above to track the distance between landmarks C and F. We also investigated the relationship between the jaws and their distance to the benthos throughout a bite. This was done by using the following equation:

3 $$d=\frac{\left|a* {x}_{{it}}+b* {y}_{{it}}+c\right|}{\sqrt{{a}^{2}+{b}^{2}}}$$

Where ${x}_{{it}}$ and ${y}_{{it}}$ are the coordinates of a landmark $i$ at time $t$, and $a$, $b$, and $c$ are the parameters of a straight line along the benthos with a formula of: ${ax}+{by}+c=0$.

We also observed some elements of behaviour which were analysed based on whether they were present or absent in each bite sequence. These included suction and whether the fish conducted a ‘pushing-off’ behaviour from the substratum (henceforth referred to as ventral expansion), prior to initiating a head flick. The presence of suction was quantified based on whether movement of the filamentous algae was detected towards the mouth of the fish when the mouth was at close proximity. The presence of ventral expansion was quantified based on the presence of cranial rotation and body elevation while the fish had its jaws closed around algae and it remained attached to the substrate.

### Biomechanical interpretation testing

Based on our results for Phases 2 and 3, we attempted to link the observed motion to the anatomy of the fish. This model (Fig. 5) consists of a motion input on the dorsal part of the fish, and a motion output on the ventral side of the fish. The dorsal flexion of the body on the head is transmitted through distinct regions of the skull and pectoral girdle, which act as output levers (see landmarks E, I and O in Fig. 5b). These output levers start at the fulcrum (rotation point between body and neurocranium), and end on the attachment points between skeletal elements (neurocranium, pectoral girdle, pelvis, postcleithrum, anal fin) and the ventral musculature (protractor hyoideus, sternohyoideus, obliquus inferioris, hypaxial). To assess the accuracy of the model described above, we compared specific motions observed in the videos, to the equivalent motions predicted based on the length of input and output levers on each specimen (i.e., anatomy). Since the specimens varied slightly in size, anatomical levers were measured on each specimen, and were the ones used in comparisons to videos from the respective specimens.

For the motion input, we compared the amount of motion carried out by landmark L due to cranial rotation (see Figs. 1e,  5a), to the amount of motion predicted, given an isosceles triangle L_ti_, K, L_tj_ (Fig. 5b) where θ_1_ is the angle change recorded from the videos. We then compared the extent of motion input (θ_1_, angle between landmarks AKL in Fig. 1e) to the extent of motion output (θ_2_, angle between landmarks EKI + IKO in Fig. 1e) from our videos. This was done to assess the motion transmission efficiency from the dorsal part of the fish to the ventral. Finally, we assessed the models’ ability to predict motion ventrally. The amount of motion observed ventrally from the videos, was the sum of distance change between landmarks EI and distance change between landmarks IO, Fig. 1e). This sum was compared to the motion predicted by using trigonometric functions on the triangle formed by landmarks EKO (see Fig. 1e), where levers EK, KO, and the amount of change in θ_2_ is known, and the motion output is defined as the sum of change between landmarks EI and IO.

### Statistical analysis

The influence of algal length on suction and ventral expansion, was tested using Generalized Linear Models (GLM), with the algal length targeted at each bite being the independent variable, and the presence/absence of the different fish behaviours being the dependent variables. Each GLM was done using a binomial distribution. We also used GLMs for the regressions comparing predictions by our biomechanical interpretations (independent variable) to the equivalent motions observed in the videos (dependent variable), and when comparing the θ_1_ angle motion input (independent variable) to the θ_2_ angle motion output (dependent variable). For these GLMs a Gaussian distribution was used. Model validation and diagnostics (e.g., qq plots, residuals, homogeneity of variance) were carried out following Zuur, et al. ^59^, using the R packages *DHARMa*^60^, *effects*^61^, and *emmeans*^62^. When comparing the duration between the first and second mouth opening in a bite (see Results) we found unequal variances, which were detected based on Levene’s test of homogeneity of variance. This analysis was therefore done with a Generalized Least Squares (GLS) model in the *nlme* package^63^, where a variance component was included, allowing the two categorical levels (1st jaw opening and 2nd jaw opening) to have different variances.

### Reporting summary

Further information on research design is available in the Nature Portfolio Reporting Summary linked to this article.

### Supplementary information

Supplemental Material

Description of Additional Supplementary Files

Supplemental Movie 1

Supplemental Movie 2

Reporting Summary

## Data Availability

All data can be found at: 10.6084/m9.figshare.24715047.v1^64^.

## Code availability

All relevant code can be found at: 10.6084/m9.figshare.24715047.v1.
