# Supplementary material for: Feeding kinematics of a surgeonfish reveal novel functions and relationships to reef substrata
Source: Commun Biol. 2024 Jan 3;7:13. doi: 10.1038/s42003-023-05696-z (PMC10764775; doi:10.1038/s42003-023-05696-z)
Supplement: Supplementary file 1 — Supplemental Material [file 42003_2023_5696_MOESM1_ESM.pdf]

Supplemental Material for: Feeding kinematics of a surgeonfish reveal novel functions and relationship to reef substrata

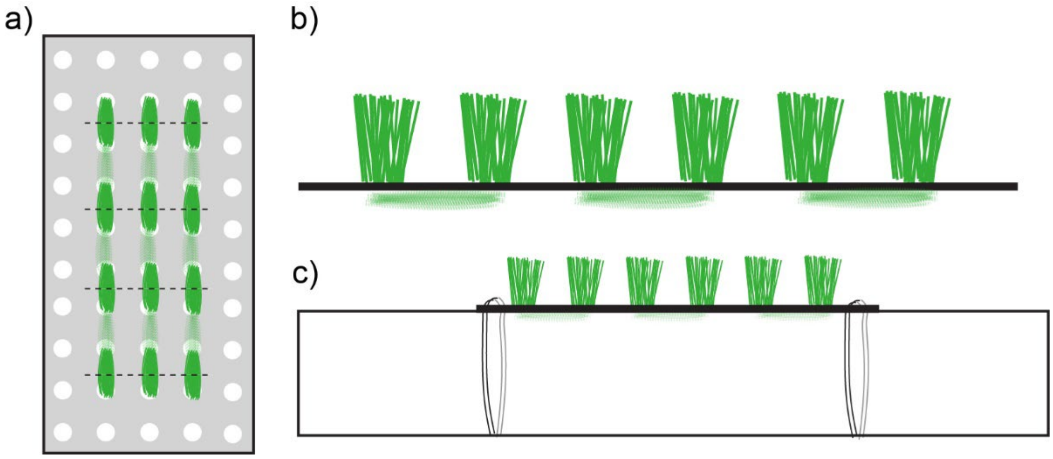

Supplemental Figure 1: Algal treatments used in our study.

Supplemental Table 1: Description of each landmark.

landmark	Description
A	Midpoint of fish nostril
B	Anteriormost point of the neurocranium
C	Anteriormost point at the tip of the upper jaw
D	Maxilla-Premaxilla joint
E	Articular-Quadrate joint
F	Anteriormost point at the tip of the lower jaw
G	Earthbound point 1: A point on the surface of the substratum at t=0, so that when a vertical line from the point is drawn, it goes through the base of the pectoral fin (landmark J)
H	Earthbound point 2: A point on the surface of the substratum (y-axis), that is one gape length away from the tip of the upper jaw (landmark C)
I	The ventralmost point on the ventral part of the fish, where the colouration switches from black to white
J	Anteriormost point of the base of the pectoral fin
K	Craniovertebral joint

**L**	Midpoint at the base of the first dorsal spine
**M**	Tip of the leading edge of the pectoral fin
**N**	Tip of the trailing edge of the pectoral fin
**O**	Midpoint at the base of the first anal spine
**P**	Dentary-Articular joint

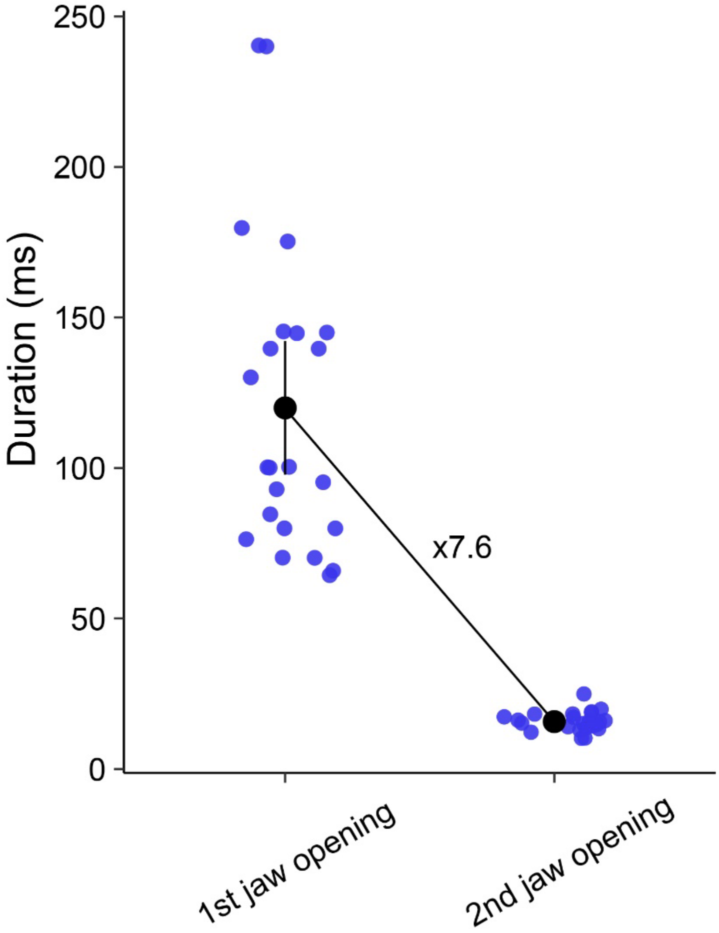

Supplemental Figure 2: Duration (ms) of the first and second jaw opening. Blue points represent raw data, whereas black points represent model results.

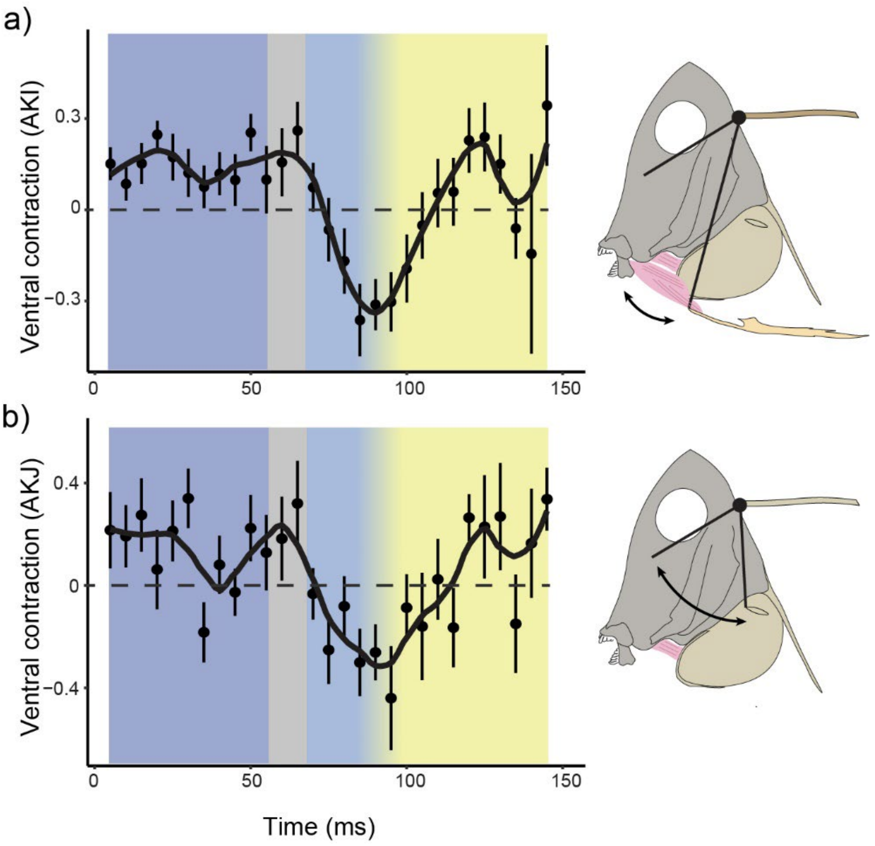

Supplemental Figure 3: Mean kinematic profiles of anatomical components for all bites (n=23). Plots show the mean amount of angular change that takes place between timesteps ($angle_t$ – $angle_{t-1}$).
