# Supplementary material for: Feeding kinematics of a surgeonfish reveal novel functions and relationships to reef substrata
Source: Commun Biol. 2024 Jan 3;7:13. doi: 10.1038/s42003-023-05696-z (PMC10764775; doi:10.1038/s42003-023-05696-z)
Supplement: Supplementary file 2 — Description of Additional Supplementary Files [file 42003_2023_5696_MOESM2_ESM.pdf]

Description of Additional Supplementary Files

File name: Supplemental Movie 1
Description: Feeding sequence of *Acanthurus leucosternon*, with each Phase noted.

File name: Supplemental Movie 2
Description: Function of the intra-mandibular joint in the surgeonfish *Acanthurus leucosternon.*
